# Advances and Unmet Needs in the Therapeutics of Bone Fragility

**DOI:** 10.3389/fendo.2018.00505

**Published:** 2018-09-06

**Authors:** Sabashini K. Ramchand, Ego Seeman

**Affiliations:** ^1^Department of Medicine, The University of Melbourne, Melbourne, VIC, Australia; ^2^Department of Endocrinology, Austin Health, The University of Melbourne, Melbourne, VIC, Australia; ^3^Mary Mackillop Institute of Health Research, Australian Catholic University, Melbourne, VIC, Australia

**Keywords:** anabolic agents, antiresorptive agents, bone fragility, fractures, microstructure, osteoporosis, osteopenia

## Abstract

The prevalence of fragility fractures increases as longevity increases the proportion of the elderly in the community. Until recently, the majority of studies have targeted women with osteoporosis defined as a bone mineral density (BMD) T score of < −2.5 SD, despite evidence that the population burden of fractures arises from women with osteopenia. Antiresorptive agents reduce vertebral and hip fracture risk by ~50 percent during 3 years but efficacy against non-vertebral fractures, 80% of all fractures in the community, is reported in few studies, and of those, the risk reduction is only 20–30%. Recent advances in the use of antiresorptives and anabolic agents has addressed some of these unmet needs. Zoledronic acid is now reported to reduce vertebral and non-vertebral fractures rates in women with osteopenia. Studies using teriparatide demonstrate better vertebral and clinical (symptomatic vertebral and non-vertebral) antifracture efficacy than risedronate. Abaloparatide, a peptide sharing amino acid sequences with teriparatide, reduces vertebral and non-vertebral fractures. Romosozumab, a monoclonal antibody suppressing sclerostin, reduces vertebral and non-vertebral fractures within a year of starting treatment, and does so more greatly than alendronate. Some recent studies signal undesirable effects of therapy but provide essential cautionary insights into long term management. Cessation of denosumab is associated with a rapid increase in bone remodeling and the uncommon but clinically important observation of increased multiple vertebral fractures suggesting the need to start alternative anti-resorptive therapy around the time of stopping denosumab. Antiresorptives like bisphosphonates and denosumab suppress remodeling but not completely. Antifracture efficacy may be limited, in part, as a consequence of continued unsuppressed remodeling, particularly in cortical bone. Bisphosphonates may not distribute in deeper cortical bone, so unbalanced intracortical remodeling continues to cause microstructural deterioration. In addition, suppressed remodeling may compromise the material composition by increasing matrix mineral density and glycosylation of collagen. As antiresorptive agents do not restore microstructural deterioration existing at the time of starting treatment, under some circumstances, anabolic therapy may be more appropriate first line treatment. Combining antiresorptive and anabolic therapy is an alternative but whether anti-fracture efficacy is greater than that achieved by either treatment alone is not known.

## Introduction

Bone remodeling, a sequential process of bone resorption and formation, occurs throughout life renewing the composition of the mineralized matrix volume (1). During young adulthood, bone remodeling is balanced—an equal volume of bone is resorbed and subsequently replaced so no net loss or gain occurs (2). Around midlife, bone formation by the osteoblasts of the basic multicellular units (BMUs) decreases, producing remodeling imbalance (3). In addition, as a consequence of the estrogen deficiency accompanying menopause, remodeling imbalance worsens and the rate of remodeling increases—less bone is deposited than was resorbed by each of the many BMUs initiated upon the three (intracortical, endocortical, trabecular) components of the endosteal (inner) bone surface (4). Estrogen therapy, by influencing the lifespan of osteoblasts and osteoclasts, may reverse the estradiol dependent component of the remodeling imbalance (5–7).

There is a reduction in total mineralized bone matrix volume and the decreasing total mineralized bone matrix volume becomes deteriorated in its microstructure. Unbalanced remodeling upon trabeculae cause them to thin, perforate and disappear. Unbalanced intracortical remodeling initiated upon the intracortical canal surfaces enlarge them, they coalesce and fragment the cortex. With advancing age bone loss from the trabecular compartment lessens because trabeculae with their surfaces disappear (remodeling requires a surface to be initiated upon). Bone loss becomes predominantly cortical as intracortical surface area increases facilitating initiation of unbalanced intracortical remodeling (8, 9). The microstructural deterioration produces bone fragility out of proportion to the bone loss producing it (10).

The burden of fragility fractures is increasing in absolute terms because longevity is increasing the proportion of the population over 65 years of age (11). Reducing the burden of fractures is an unmet need because there are unresolved issues in detection of individuals at high risk for fracture that need to be addressed (12). For example, identifying methods able to detect individuals at imminent risk for fracture is a challenge. Commonly used tools such as bone densitometry lack sensitivity. A BMD T score threshold of − 2.5 SD designated as “osteoporosis” identifies only 30–40% of women having fragility fractures (13–15).

The word “osteoporosis” is often used synonymously with bone fragility but women with osteopenia are not free of the risk of fracture (13, 15). Indeed, most women and men sustaining fragility fractures have osteopenia and many have so-called “normal” BMD (14). Women with osteopenia at risk for fracture can be identified by measuring microstructural deterioration (16, 17) but high-resolution imaging methods are not yet widely available. The use of clinical risk factor assessment tools such as FRAX have met with variable success (18, 19). Challenges also arise in the uptake and adherence to therapy, in part, because of concerns regarding the serious but uncommon long term adverse effects of therapy (20, 21).

Antiresorptive agents are the first line and most commonly used treatments for prevention and treatment of bone fragility (22). Apart from denosumab, which virtually abolishes remodeling, most antiresorptives slow unbalanced remodeling so microstructural deterioration continues to occur albeit more slowly (23). This lower rate of remodeling reduces fracture risk compared to untreated women in whom rapid remodeling continues to deteriorate the skeleton. This is a relative risk reduction. In absolute terms, fracture risk does not decrease during antiresorptive therapy because microstructural deterioration present is not reversed and the slow continued unsuppressed and unbalanced remodeling continues to deteriorate bone. This, in part, may explain why fracture risk reduction with antiresorptives is modest. Teriparatide increases bone matrix volume predominantly through remodeling based bone formation (24). It is likely that the anabolic effect of abaloparatide, which acts via the same receptor as teriparatide, is also remodeling based like teriparatide, although rigorous assessment of its mechanism of action has not been undertaken (25). Both reduce the risk of vertebral and non-vertebral fractures (26, 27) but no adequately designed trials have been done to determine whether hip fracture risk is reduced.

Several comprehensive literature reviews are available (28, 29). We confine this manuscript to defining advances that have taken place in existing therapies and new therapies that are available or may become available soon, particularly the development of anabolic agents, therapies that partly reconstruct the skeleton but are also not without their limitations.

## Anti-resorptive therapy

### Bisphosphonates

Bisphosphonates are currently first line treatment and the most common antiresorptive therapy used. The antiresorptive efficacy of bisphosphonates depend on inhibition of farnesyl pyrophosphate (FPP) synthase, required for osteoclast resorptive function, as well as their affinity for mineral which influences uptake, distribution, and retention in the bone (30–33). High affinity binding agents, like alendronate, have a reduced ability to penetrate and distribute widely in deeper cortical matrix so that when osteoclasts remodel cortical bone they encounter matrix free of bisphosphonates and continue to resorb bone.

This may partly account for the finding of less reduction in porosity with alendronate than denosumab (34). The reduction in porosity is the net result of fewer cavities being excavated plus the incomplete filling of the resorption cavities excavated shortly before treatment (35). Likewise, ibandronate is avidly bound to matrix mineral and is present in lower concentrations in cortical than trabecular bone. Studies of ibandronate in ovariectomized cynomolgus monkeys demonstrate reduced remodeling and improved trabecular bone strength but not intracortical remodeling suppression or improved cortical bone strength (36). Lower binding affinity of risedronate than alendronate results in wider distribution of risedronate through the bone and may contribute to its earlier suppression of bone remodeling as reported in animal models and claims of possible earlier fracture risk reduction (37, 38).

Several advances have been made in the study of zoledronic acid. This drug is usually given as an annual 5 mg infusion for 3 years. Fracture risk is reduced in postmenopausal women with osteoporosis, by 77% for clinical (symptomatic) vertebral fractures, 25% for non-vertebral, and 41% for hip fractures (39). Zoledronic acid was also associated with a 28% reduction in mortality after hip fracture, independent of its effects on fracture risk reduction (40, 41).

Discontinuation of zoledronic acid at 3 years, followed by no treatment for 3 years, was associated with minimal reduction in BMD but 30 new morphometric vertebral fractures occurred compared to 14 in those treated for 6 years (odds ratio = 0.51; *P* = 0.035) (42) In further follow-up, women treated for 6 years and 3 years off treatment did not have more fractures than women treated for 9 years. However, the loss of the inception cohort and small numbers of events make interpretation problematic (43).

Several very insightful studies have been published regarding zoledronic acid treatment. In a *post-hoc* analysis of pooled data from the HORIZON studies, there were comparable ~30% reductions in clinical fractures in those who received a single infusion or three or more annual infusions (44). Whether differing baseline characteristics influenced this outcome is not clear, but given the protracted remodeling suppression with this drug, it is of interest to determine the appropriate regimen needed for efficacy and safety.

This has been evaluated by Reid et al. in postmenopausal women with osteopenia randomized to a single dose of zoledronic acid 1, 2.5, or 5 mg. These doses resulted in a similar increase in spine and hip BMD in the 2.5 and 5 mg groups at 2 years but not at 5 years (45), where increases were greater in the 5 mg than 2.5 mg group (46).

More recently, Reid et al. addressed two important unmet needs. There is a lack of information regarding the antifracture efficacy of drugs given to women with osteopenia, the source of over 60% of all fragility fractures (13). There is also little information regarding the prevention of non-vertebral fractures, 80% of all fractures in the community (11). The investigators evaluated 2,000 women with osteopenia, mean age 71 years, treated with zoledronic acid 5 mg or placebo every 18-months for 4 doses. After 6 years of follow up, treated women had a 34% reduction in non-vertebral fractures (HR 0.66, 95% CI 0.51–0.85) (47).

### Denosumab

Denosumab is a fully humanized monoclonal antibody directed against RANK-ligand, a major regulator of osteoclast development which inhibits osteoclast recruitment, activity and survival. Denosumab (60 mg subcutaneously administered every 6 months) produces almost complete suppression of bone remodeling. Treatment for 3 years resulted in a 68% reduction in vertebral fractures, 40% reduction in hip fracture and 20% reduction in non-vertebral fractures (48).

Remodeling suppression with denosumab is greater than that achieved with any other antiresorptive agent (49). This greater suppression of remodeling accounts for greater increases in BMD achieved in postmenopausal women treated with denosumab compared to bisphosphonates (34, 50–52). Whether these BMD differences translate to differences in fracture risk reduction between the two groups is not known.

No trials have been conducted with a placebo control group beyond 3 years. Because of this, whatever the fracture rate, it is not possible to infer with confidence that fracture rates reported are attributable to the drug rather than healthy user bias. In the open label extension of the FREEDOM study (53), all participants were treated with denosumab and followed up for 7 years. Ten years of denosumab treatment was associated with an acceptable safety profile, sustained suppression of bone remodeling, continued increase in BMD without plateau and “low” fracture rates. The lack of controls and the large number of participants withdrawn from the trial, suggest that the finding of low and further reductions in fracture rates should be cautiously interpreted.

In the long-term group, BMD increased by 21.7% in the lumbar spine and 9.0% in the femoral neck at 10 years compared to FREEDOM baseline. The mechanism of increase in BMD may be due to progressive secondary mineralization. It is plausible that in the face of suppressed remodeling, continued slow age-related modeling based bone formation becomes detectable as reported in studies of histomorphometry conducted in cynomolgus monkeys (54, 55). Dempster et al. also report some evidence of modest trabecular modeling based bone formation after 3 months of denosumab therapy (56). Further studies are needed to determine whether antiresorptive therapy permits expression of any existing modeling based bone formation when remodeling is suppressed.

An important insight into treatment with denosumab is the report that cessation of denosumab caused a rapid rise in bone remodeling markers with a transitory overshoot above baseline, a decline in BMD in 12 months and, uncommonly, an increased risk in multiple vertebral fractures (57, 58). Occurrence of multiple vertebral fractures were initially reported in several case series (59–64). In a subsequent *post hoc* analysis of the FREEDOM and FREEDOM Extension trials (65), vertebral fracture rate after discontinuation of denosumab increased to 7.1 per 100 participant years vs. 8.5 per 100 participant years in the placebo group. In those who developed one or more vertebral fractures, the proportion of multiple vertebral fractures (>2) was higher in those discontinuing denosumab (60.7%) than in those discontinuing placebo (38.7%, *P* = 0.049). The risk of multiple vertebral fractures after stopping denosumab was greatest in those with a prior vertebral fracture, either before or during treatment (odds 3.9, 95% CI 2.1–7.2) (65). Investigators recommend commencement of an alternative antiresorptive agent soon after cessation of denosumab, although the agent to use and when to start needs further evaluation.

At present, there is no evidence that the increased risk of vertebral fractures is due to the rapid increase in remodeling. The term “rebound” is used but cessation of remodeling suppression produces expansion of the reversible remodeling space with any antiresorptive (66). When an antiresorptive is stopped, the effect has similarities to the onset of menopause. There is a rapid increase in the number of remodeling units starting to excavate bone and hence a rapid rise in remodeling markers. With denosumab, given the drug is not retained in the skeleton like bisphosphonates, the increased number of resorptive cavities is not offset as it is with bisphosphonate cessation. With bisphosphonate cessation, increased resorption may lead to release of bisphosphonate and readsorption into matrix slowing the decrease in BMD. In the absence of denosumab, the rapid increase in number of resorption cavities is not inhibited and may create stress concentrators which pre-dispose to microcrack propagation and increased vertebral fracture risk (67, 68). Whether this increase in resorption is amplified by rapid differentiation of existing osteoclast precursors remaining undifferentiated until denosumab is stopped is not known.

While this “rebound” or “overshoot” in remodeling markers is reported, remodeling returns to its pretreatment level and the reduction in BMD returns to baseline leaving a residual higher BMD than untreated controls (58, 69). If there was accelerated loss of bone, beyond that found early after stopping treatment due to many more excavated cavities than those incompletely refilling after starting denosumab, then BMD should decline to levels no different to untreated controls (70). Bone fragility is likely to occur after stopping treatment because the accelerated remodeling occurs in the setting of an already deteriorated skeleton (as antiresorptive agents do not reverse microstructural deterioration present at the time of starting treatment). This is not the same after menopause where there is little, if any, deterioration before menopause.

### Selective estrogen receptor modulators

Raloxifene, a selective estrogen receptor modulator (SERM), reduces the rate of bone remodeling by about 20–30% as determined using circulating bone remodeling markers (71). It produces a modest transitory increase in BMD during early treatment, as fewer new resorption cavities are excavated while the many more cavities excavated before treatment refill, albeit incompletely. With more protracted treatment, unbalanced remodeling continues at 20–30% slower rate than before treatment so microstructural deterioration continues (70). The decline in BMD during prolonged therapy is well documented (71, 72) and probably accounts, in part, for the modest vertebral fracture risk reduction and lack of evidence for non-vertebral fracture risk reduction (71).

Raloxifene appears to reduce vertebral fractures with small and perhaps transient effects on BMD. It is of interest that pre-clinical studies demonstrate an increase in the material strength produced by increases in skeletal-bound water with minimal effect on tissue mineral composition or microdamage accumulation (73–75). These findings provide a novel target for future pharmacological interventions to improve bone strength and lower fracture risk. This is of particular interest given the concerns of protracted remodeling suppression by bisphosphonates and denosumab which are likely to pre-dispose to loss of toughness and atypical femoral fractures (AFFs).

Treatment of osteoporosis in patients with AFFs is challenging; withholding an antiresorptive will result in ongoing structural decay predisposing to fragility fracture, conversely, if an antiresorptive is continued, structural decay will slow but material composition will be compromised predisposing to AFFs (76). One approach may be to use a weaker antiresorptive such as raloxifene, which improves bone toughness with minimal effects on tissue mineral composition or microdamage accumulation. The efficacy of raloxifene in this context has not been established.

## Anabolic agents

Teriparatide (PTH 1-34) and abaloparatide are available for clinic use, romosozumab is a modeling based anabolic agent that is still under investigation.

### Teriparatide and abaloparatide

The anabolic effects of PTH 1-34 are ~70% remodeling based. Abaloparatide, which shares amino acid sequences with both parathyroid hormone-related protein (PTHrP) and PTH, acts via the same receptor as teriparatide (PTHR1). Both teriparatide and abaloparatide increase trabecular thickness and improve trabecular microstructure (77–79). There is a transitory phase of increased cortical porosity produced with PTH 1-34 (80, 81). Whether the anabolic effect of abaloparatide is accompanied by less resorptive activity and less cortical porosity needs to be confirmed (27, 82, 83).

Parathyroid hormone analogs have consistently been reported to reduce vertebral fractures but evidence for non-vertebral fracture risk reduction reported by Neer et al. has not been replicated (84). No randomized controlled studies have been done to evaluate anti-hip fracture efficacy, an omission that needs to be addressed.

Abaloparatide also reduces vertebral and non-vertebral fractures (27). In a phase 3 clinical trial, 2463 ambulatory postmenopausal women, of which 1901 completed the study, were randomized to 18 months of abaloparatide (80 μg daily), placebo or open-label teriparatide (20 μg daily). New morphometric vertebral fractures occurred in 0.58% (*n* = 4) of the abaloparatide group, 4.22% (*n* = 30) of the placebo group (relative risk 0.14, 95% CI 0.05–0.39), and 0.84% (*n* = 6) of the teriparatide group. The Kaplan-Meier estimated event rate for non-vertebral fracture was 2.7% for abaloparatide, 4.7% for placebo (HR 0.57, 95% CI 0.32–1.00), and 3.3% for teriparatide (*P* = 0.22 compared to placebo and *P* = 0.44 compared to abaloparatide) (27). Major osteoporotic fractures were reduced with abaloparatide compared to placebo or teriparatide, Kaplan-Meier estimated event rate for placebo was 6.2% (HR 0.30, 95% CI 0.15–0.61) and for teriparatide was 3.1% (HR 0.45, 95% CI 0.21–0.95, *P* = 0.03) (27).

While these findings are encouraging, the claim that abaloparatide produced an earlier and more efficacious fracture risk reduction than teriparatide is problematic because of an increase in number of subjects who fractured in the first few weeks of treatment in the placebo and teriparatide group that is unlikely to be associated with therapy (27). Differences in fracture rates in the two treatment arms in the second and third 6 months of the 18-month trial were minimal (83). In addition, evidence that the anabolic effect is accompanied by less bone resorption with abaloparatide than teriparatide is also not well founded (83).

Increases in BMD with abaloparatide were greater (~1%) than those with teriparatide at the total hip and femoral neck at all time points and ~2% at the lumbar spine at 6 and 12 months (both *P* < 0.001). At 18-months, BMD in the lumbar spine was no different between the two groups (27). These difference in BMD at the femoral neck and total hip were attributed to the net difference in resorption and formation markers claimed to be surrogates of more net bone deposition with abaloparatide than teriparatide, a problematic interpretation for a range of reasons (83, 85).

### Romosozumab

Romosozumab is a humanized monoclonal antibody against sclerostin, an endogenous inhibitor of bone formation. Treatment results in an increase in modeling based bone formation and evidence of decreases in bone resorption. Two studies support the antifracture efficacy of this anabolic agent (86, 87). Cosman et al. enrolled 7,180 postmenopausal women with osteoporosis to monthly romosozumab (210 mg) or placebo for 12 months followed by denosumab (60 mg 6 monthly) for 12 months (86). At 12 months, risk reductions were reported for vertebral fractures by 73% (*P* < 0.001), for clinical fractures by 36% (*P* = 0.008) and non-vertebral fractures by 24% (*P* = 0.10). At 24 months, vertebral fracture risk was reduced by 75% (*P* < 0.001) (86).

Saag et al. (87). assigned 4,093 postmenopausal women with osteoporosis and a fragility fracture to romosozumab (210 mg) or weekly alendronate (70 mg) for 12 months then open label alendronate in both groups. Over 24 months, romosozumab/alendronate reduced vertebral fracture risk by 48% (*P* < 0.001), clinical fractures by 27% (*P* < 0.001), non-vertebral fracture by 19% (*P* = 0.04), and hip fracture by 38% (*P* = 0.02). At 12 months, romosozumab reduced new vertebral (risk ratio 0.63, 95% CI 0.47–0.85) and clinical (HR 0.72, 95% CI 0.54–0.96) fractures compared to alendronate. Non-vertebral fracture risk was also reduced by 26% with romosozumab, but this difference was not statistically significant (*P* = 0.06) (87). The use of romosozumab is under FDA review after results of the trial demonstrated a higher incidence of adjudicated serious cardiovascular events with romosozumab (50/2040) compared to alendronate (38/2014) at the end of 12 months, which did not persist in the 24-month open label extension (87). These findings were not replicated in the much larger placebo-controlled FRAME study (88).

Recent work by McClung et al. (89). report loss of benefit of romosozumab soon after cessation of therapy. Three hundred and sixty-four postmenopausal women with low bone mass were treated with romosozumab for 24 months and then randomized to either denosumab or placebo for a further 12 months. Treatment with romosozumab led to a continued increase in BMD over 2 years with further accrual in those that transitioned to denosumab, whereas BMD returned toward pre-treatment levels in those that transitioned to placebo (89).

Given the inability of antiresorptives to reverse existing microstructural deterioration, and the evidence that anabolic therapy may partly restore bone microstructure, is there evidence supporting better antifracture efficacy using anabolic therapy than antiresorptive therapy. Kendler et al. studied 1360 postmenopausal women with severe osteoporosis randomized to teriparatide (20 μg daily) or risedronate (35 mg daily) over 2 years (26). Overall, 72% of participants received at least one bone targeted treatment prior to study entry, most commonly a bisphosphonate (59% in the teriparatide group and 57% in the risedronate group) and median duration of bisphosphonate treatment was 3.5 years (IQR 1.1–7.0) in the teriparatide group and 3.6 years (IQR 1.3–6.1) in the risedronate group. At 2 years, treatment with teriparatide resulted in a 56% (risk ratio 0.44, 95% CI 0.29–0.68) reduction in incident vertebral fractures with a reduction in non-vertebral fractures that did not achieve statistical significance; 25 (4.0%) in the teriparatide group vs. 38 (6.1%) in the risedronate group (hazard ratio (HR) 0.66, 95% CI 0.39–1.10, *P* = 0.10) (26). In a subgroup analyses, these changes were consistent across a range of characteristics of the participants (90).

### Combined antiresorptive and anabolic therapy

Combining antiresorptive and anabolic therapy is a missed opportunity for two reasons (70). First, no studies have been done demonstrating greater antifracture efficacy than achieved by either treatment alone. This is a valid reason for a cautionary approach to the uptake of this regimen. The second reason is the widely held belief that antiresorptive therapy suppresses, “blunts,” remodeling based bone formation by PTH (91–93). This is largely based on two influential papers and the accompanying editorial in the New England Journal of Medicine (91, 93, 94). The notion of blunting was based on the assumption that a higher BMD or higher P1NP mean more bone formation and a lack of response means less bone formation.

Comparator studies that use changes in BMD and bone remodeling markers as the outcome variable are problematic endpoints. Remodeling based anabolic therapy increases bone matrix volume by replacing more fully mineralized bone with young less fully mineralized bone. Modeling based anabolic therapy adds young less fully mineralized bone to existing older bone. Imaging using radiation transmission often results in a net reduction in BMD because young less mineralized bone transmits rather than attenuates photons leading to the inference that bone “loss” and fragility have occurred. Antiresorptives slow remodeling. Matrix no longer “turned over” undergoes more complete mineralization increasing BMD leading to the inference that bone “volume” or “mass” has increased, and that bone strength has increased even though the matrix becomes less ductile.

As an example, even if an increase or lack of an increase in BMD is accepted on face value, examination of Figures 1 to 3 of the study by Black et al. does not support the notion of blunting (91). Relative to PTH alone, combined therapy (i) did *not* produce a smaller increment in spine or femoral neck BMD, (ii) *did* produce a greater increase in total hip BMD, (iii) *did* reduce the decline in distal radius BMD, (iv) *did* prevent the reduction in total hip and femoral neck vBMD produced by PTH alone. Curiously, the increase in total hip and femoral neck cortical volume by PTH, a *modeling* effect, was prevented by combined therapy. Moreover, combined therapy increased trabecular vBMD less than PTH alone but this may be a benefit, not blunting. The antiresorptive might prevent PTH mediated increase in intracortical remodeling, cortical porosity and the increase in cortical fragments that look like “trabeculae” (35). Blunting of the rise in P1NP and CTX is likely to be the result of suppressed remodeling, not a reduction in the net volumes of bone deposited or resorbed respectively (85). If blunting of the BMD response was due to fewer BMUs then blunting should be *more* severe with co-administration of PTH with zolendronate, denosumab, or osteoprotegerin (OPG, an endogenous inhibitor of RANKL) than with alendronate. The opposite is reported, and many studies report additive effects (95–98).

The difficulties in using BMD are also present using high-resolution peripheral computed tomography. Tsai et al. (80). report that combined PTH 1-34 and denosumab increased cortical vBMD yet PTH 1-34 reduced it and denosumab had no effect. Combined therapy increased cortical matrix mineral density yet PTH 1-34 decreased it and denosumab had no effect. Combined therapy had no effect on porosity yet PTH 1-34 increased it while denosumab had no effect. These findings do not add up, probably because there are methodological challenges in segmenting (separating) the cortical and trabecular compartments and quantifying porosity and trabecular density because low image resolution and changes in matrix mineral density influence the quantification of microstructure (8, 99).

### Sequential therapy

#### Anabolic to anti-resorptive

Cessation of anabolic treatment results in loss of the benefits. Antiresorptives maintain or increase BMD, particularly denosumab because it is the most efficacious in suppressing remodeling. In the DATA-Switch study, 2 years of PTH 1-34 followed by 2 years of denosumab resulted in further increases in BMD (100). At 48 months, women treated with combined PTH 1-34 and denosumab for 2 years followed by denosumab alone had greater gains in BMD than those treated with PTH 1-34 followed by denosumab. Whether this results in fewer fractures is not known but it is likely that stopping any of the treatments will result in the loss of benefits and eventual increase in fracture risk.

In another publication of the abaloparatide trial by Miller et al. (27), Bone et al. (101) administered alendronate after abaloparatide which maintained the fracture risk reduction relative to placebo also given alendronate after 18 months. This design does not address the question of whether stopping abaloparatide produces loss of benefits as found with PTH, which requires an arm with abaloparatide given placebo. Any comparisons of abaloparatide/alendronate and placebo/alendronate is flawed as the placebo group is likely to have undergone bone loss and microstructural deterioration during 18 months. The likelihood is however, that stopping abaloparatide will result in loss of benefits. This has recently been reported with romosozumab; where stopping treatment was accompanied by loss of the benefits achieved by the modeling dependent anabolic effect (89).

#### Anti-resorptive to anabolic

Two recent trials evaluating antiresorptive therapy followed by an anabolic agent have been conducted (100, 102). In the DATA-switch study, women receiving denosumab had a reduction in hip BMD during 12 months of PTH 1-34 followed by a gradual increase in BMD, but remained lower than women in the PTH 1-34 to denosumab group and the PTH 1-34/denosumab to denosumab group (100). Spine BMD decreased in the first 6 months but then increased to a value no different to the above two groups at 48 months. Bone remodeling markers increased in the denosumab to PTH 1-34 group by over 200% relative to baseline values. Whether bone fragility increases is difficult to determine given the decline in BMD may be due to the replacement of more mineralized with less mineralized new bone by remodeling or addition of under mineralized bone by modeling which then becomes mineralized. High remodeling is found using anabolic agents but fracture rates do not increase, they decrease. Nevertheless, cessation of denosumab is associated with rapid increases in remodeling and multiple vertebral fractures; whether this might be prevented or worsened by PTH 1-34 is not known.

In an unblinded study comparing romosozumab (120 mg monthly) vs. PTH 1-34 (20 μg daily) in postmenopausal women previously treated with bisphosphonates, total hip BMD increased with romosozumab by 2.6% compared to a decrease of 0.6% with PTH 1-34 (102). Both drugs increased spine BMD (romosozumab 9.8% vs. PTH 1-34 5.4%). At the hip, romosozumab increased cortical vBMD whilst PTH 1-34 decreased it. Trabecular vBMD was similarly increased with both drugs (102). The comparison of BMD changes is problematic given BMD may decrease when a large volume of bone that is still unmineralized is deposited and the effects on microstructure which contribute disproportionately to bone strength are not taken into account.

## Conclusion

Fragility fractures are a public health burden. Advances are occurring, but several challenges remain unmet. Most fractures occur in women with osteopenia yet methods of identifying the women forming the population burden of fractures remain to be identified. Even when women at risk are identified, the uptake and adherence to therapy is poor for reasons that are not well defined. Antiresorptive agents are first line approaches to therapy even though these agents do not restore bone volume or the microstructural deterioration present at the time of treatment. Most, if not all, controlled trials are 3 years duration and long-term efficacy is unknown. Anabolic therapies have not been as comprehensively studied. Although newer agents are emerging and vertebral fracture risk reduction is confirmed, less evidence is available for non-vertebral fracture risk reduction, and no anabolic agent has been evaluated for hip fracture risk reduction in randomized controlled studies. Bone densitometry was a good beginning but most fractures in the community arise in persons with a BMD T-score less reduced than −2.5 SD and so they are not offered treatment. Whether assessment of bone microstructure might help identify and target therapy more effectively remains an unmet challenge, but it is an opportunity in need of exploration because bone fragility is caused by microstructural deterioration.

## Author contributions

All authors listed have made a substantial, direct and intellectual contribution to the work, and approved it for publication.

### Conflict of interest statement

ES has received research support and lecture fees from Amgen, Allergan, and Eli Lilly; he is a director of the board and shareholder of StraxCorp. The remaining author declares that the research was conducted in the absence of any commercial or financial relationships that could be construed as a potential conflict of interest.
